# Recognition of Human Activities Using Depth Maps and the Viewpoint Feature Histogram Descriptor

**DOI:** 10.3390/s20102940

**Published:** 2020-05-22

**Authors:** Kamil Sidor, Marian Wysocki

**Affiliations:** 1 Section of Informatization of the Course of Studies, Rzeszow University of Technology, al. Powstancow Warszawy, 12 35-959 Rzeszow, Poland; 2 Department of Computer and Control Engineering, Faculty of Electrical and Computer Engineering, Rzeszow University of Technology, W. Pola 2, 35-959 Rzeszow, Poland; mwysocki@prz.edu.pl

**Keywords:** point clouds, VFH descriptor, activity recognition, dynamic time warping, BiLSTM, transfer learning, multiple network fusion

## Abstract

In this paper we propose a way of using depth maps transformed into 3D point clouds to classify human activities. The activities are described as time sequences of feature vectors based on the Viewpoint Feature Histogram descriptor (VFH) computed using the Point Cloud Library. Recognition is performed by two types of classifiers: (i) k-NN nearest neighbors’ classifier with Dynamic Time Warping measure, (ii) bidirectional long short-term memory (BiLSTM) deep learning networks. Reduction of classification time for the k-NN by introducing a two tier model and improvement of BiLSTM-based classification via transfer learning and combining multiple networks by fuzzy integral are discussed. Our classification results obtained on two representative datasets: University of Texas at Dallas Multimodal Human Action Dataset and Mining Software Repositories Action 3D Dataset are comparable or better than the current state of the art.

## 1. Introduction

One of the most important tasks of human-computer interfaces is the interpretation of people’s behavior. Video systems play a central role here. Currently, solutions using modern RGB-D cameras ―which in addition to traditional images give information about depth―are becoming more and more popular. One of the best known devices for acquiring depth maps is the Microsoft Kinect^TM^ sensor. There are also other cameras of a similar type e.g., time-of-flight cameras (ToF), which are becoming cheaper and therefore more accessible.

The increase in the popularity of the Kinect^TM^ and ToF cameras has greatly contributed to an increased interest in using depth maps. This data can be considered as an aid, but also as the main source of information. Using them facilitates the separation of objects from the background, especially in poor lighting, and can support object recognition by introducing features based on 3D shape descriptors.

3D data are used, among other applications, in the recognition of static images and image sequences. Static depth images are used, for example, in [1] to recognize finger alphabet letters and in [2] for object identification. The sequences of depth images find their application in tracking people [3], as well as in recognition of peoples’ activities, e.g., [4,5,6].

In this paper we propose a way of using depth maps transformed into 3D point clouds to classify human activities. We describe the activities as time sequences of feature vectors based on the Viewpoint Feature Histogram descriptor (VFH) computed using the point cloud library (PCL) [7]. We use two types of classifiers: (i) k-NN nearest neighbors classifier with Dynamic Time Warping (DTW) and (ii) bidirectional long short-term memory (BiLSTM) deep learning networks. We use two representative datasets: the University of Texas at Dallas Multimodal Human Action Dataset [8,9], as well as the Mining Software Repositories Action 3D Dataset [10], to examine classification effectiveness and compare it with known literature results.

The paper is structured as follows: Section 2 analyses the related work and characterizes the contributions of this paper. Section 3 presents the VFH descriptor. Section 4 briefly describes the classification methods. Section 5 describes the datasets used. Section 6 gives details of the proposed recognition system. Section 7 presents the results of our experiments. Finally, the conclusions and some directions for further research are presented in Section 8.

## 2. Related Work and Contribution

There are many approaches in the literature that use depth data to recognize human activity. In [11] Wanging et al. used action graphs, where each node represents an attitude in the sequence. Vieira et al. [12] represented sequences of depth maps in short films by so called Space–Time Occupancy Patterns, where time and space are divided into segments. A modification of this approach, based on local features and called random occupancy patterns was proposed by Wang et al. [13]. The method can be helpful when depth maps do not have much texture, are noisy, or occlusions are present. Yang et al. extended in [14] the known Motion History Image method by introducing Depth Motion Maps that accumulate sequences of depths and histograms of oriented gradients. Chen et al. in [15] and in [6] used the idea of depth motion maps with some modifications and reduced computation cost by introducing a collaborative representation classifier. Oreifej et al. [16] introduced the concept of histogram of oriented 4D normals and represented the depth sequence as a histogram in 4D space. Kim et al. [17] generated side and front views of the depth map, transformed these views into descriptors of depth motion appearance and depth motion history, and used a Support Vector Machine(SVM) classifier based on these descriptors.

Another important approach to action recognition is based on 3D skeletons. In [18] the front view of the skeleton trajectory, as well as top and side views generated by rotating the 3D front viewpoints are processed by three convolutional neural networks (CNNs) for feature extraction and classification. Feature extraction and classification with CNNs are also used in [19] and [20]. The solutions are based on a descriptor representing the motion of body joints and the spatiotemporal information of a skeleton sequence encoded into color texture, respectively.

Wang et al. [21] proposed a joint descriptor, which takes into account not only the joint position but also the local space around it, and the Fourier temporal pyramid―motivated by the spatial pyramid―as a joint motion representation. The authors of [22] combined information about static posture and motion by introducing the concept of EigenJoints – features determined using differences of joints’ positions. For efficient 3-D joint features representation [23] proposes a method based on sparse coding and temporal pyramid matching. An extended summary of these descriptions is given in Table 1.

This paper presents a new method for recognizing human activities. The method is based on point clouds and the VFH descriptor. Such an approach is inspired by the coauthored works considering a specific kind of activity of deaf people speaking sign language. In this case hand shape and motion play the most important role. In [1] we used a publicly available American finger alphabet dataset [24]. This challenging dataset consists of 24 hand postures representing the letters performed a variable number of times by five people. For the classification, we used 400 depth maps for each gesture performed by each person. The results were obtained using leave-one-subject-out 5-fold cross-validation tests. Our approach turned out to be better than or comparable to other published methods. We also showed that the hand shape representation based on such an approach can also be applied for the recognition of fingerspelling considered as quick highly coarticulated motions.

In [4], based on similar method, we considered recognition of Polish Sign Language words. The experiments were carried out on our datasets containing gestures performed by an interpreter from The Polish Association of the Deaf. The gestures were acquired using a MESA Swiss Ranger 4000 ToF camera (from the Swiss Center for Electronics and Microtechnology, Zürich, Switzerland), and Mirosoft Kinect^TM^ sensor to obtain depth data. For the ToF camera, 84 Polish Sign Language words were repeated 20 times at three orientations of the gesticulating person with respect to the camera. For the Kinect^TM^ device, 30 words were repeated ten times. Words are characterized by different speeds of execution, hands are often not the objects nearest the camera, they touch each other, touch the head or appear in the background of the face. Moreover, the orientation of the person with respect to the camera is variable. The ten-fold cross-validation recognition rates about 80% are promising.

The considerations in this paper are the next step showing applicability of using point clouds and the VFH descriptor. Here we are focusing on activities engaging other body parts. The activities are registered in two representative datasets: the UTD-MHAD [9] dataset and the MSR Action 3D dataset [10]. The results related to these classes of activities obtained by our method are original. Complementing the mentioned applications related to hand gestures, these results can be seen as an argument for using the VFH point cloud descriptors for people’s activity recognition.

The contributions of this paper lay in:

- Proposition of an approach for recognition of activities with using sequences of point clouds and the VFH descriptor.
- Verification of the method on two representative, large datasets using k-NN and BiLSTM classifiers.
- Reduction of classification time for k-NN by introducing a two-tier model.
- Improvement of BiLSTM-based classification via transfer learning and combining multiple networks by fuzzy integrals.

## 3. Viewpoint Feature Histogram (VFH)

In the article the VFH descriptor was used for extracting features from depth maps. VFH is the global descriptor of a point cloud – a data structure representing a multidimensional set of points in a clockwise coordinate system [25]. The system’s *x*-axis is horizontal and directed to the left, the *y*-axis runs vertically and faces up, the *z*-axis coincides with the optical axis of the camera and is turned towards the observed objects. VFH consists of two components: a surface shape component and a viewpoint direction component. The descriptor is able to detect subtle variations in the geometry of objects even for untextured surfaces.

**Figure 1:**
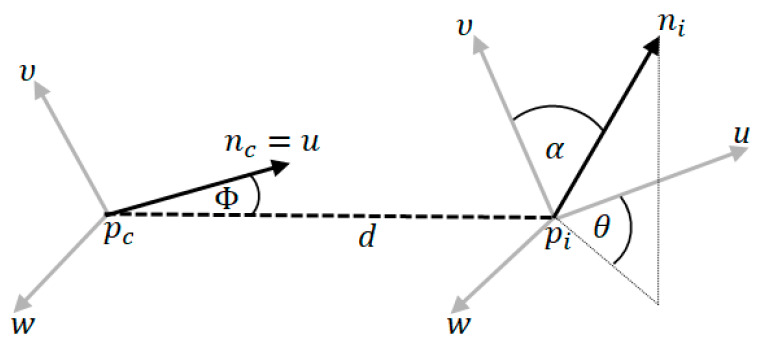
Values of the surface shape component of the Viewpoint Feature Histogram (VFH).

The first component consists of values θ,cos(α),cos(ϕ)  and d  measured between the gravity center $p_{c}\;$ and every point $p_{i}$ belonging to the cloud. $n_{c}$ is the vector with initial point at $p_{c}$ with coordinates equal to the average of all surface normals. $n_{i}$ is the surface normal estimated at point $p_{i}$. The angles θ and α can be described as the yaw and pitch angles between two vectors while d denotes the Euclidean distance between $p_{i}$ and $p_{c}$. The vectors and angles shown in Figure 1 are defined as follows [4,26]:

(1) $$u=n_{c},$$

(2) $$\upsilon =\frac{p_{i}-p_{c}}{d}\times u,$$

(3) w=u×υ,

(4) $$\cos\left(\alpha \right)=\upsilon \cdot n_{i},$$

(5) $$\cos\left(\Phi \right)=u\cdot \frac{p_{i}-p_{c}}{d},$$

(6) $$\theta =arctg\left(\frac{w\cdot n_{i}}{u\cdot n_{i}}\right),$$

**Figure 2:**
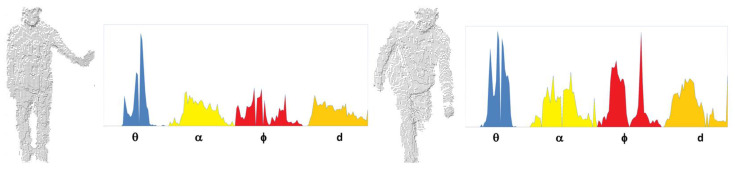
VFH histograms generated for point clouds representing two body postures.

Default histograms consist of 45 bins for each feature of the surface shape component and 128 for the viewpoint component (308 bins in total). The more detailed descriptions of VFH calculation are presented in [25,26]. A sample illustration of VFH histograms is shown in Figure 2. In the sequel we will simply use α and ϕ instead cos(α), cos(ϕ).

## 4. Classification

The means and standard deviations of the histograms obtained as VFH descriptors are used as features for classifiers. The activities analyzed are dynamic, so their feature vectors obtained for individual video frames form time series. Two types of classifiers are considered in this paper: (i) k-NN based on DTW measure, (ii) BiLSTM. DTW and BiLSTM are briefly described in the next subsections.

### 4.1. DTW

The main aim DTW is to compare two different features $X≔\left(x_{1},x_{2},\ldots ,x_{N}\right)$ of length N∈ℕ and $Y≔\left(y_{1},y_{2},\ldots ,y_{M}\right)$ of length M∈ℕ with elements sampled at equidistant time points. Feature space is denoted by ℱ, then $x_{n},y_{m}\in ℱ$ for n∈[1:N] and m∈[1:M]. To compare two different features x,y∈ℱ, one needs a local distance measure, which is defined to be a function [27]:

(7) $$c\;:ℱ\times ℱ\;\to \;{\mathbb{R}}_{\geq 0}$$

If *x* and *y* are similar, the value of c(x,y) epresenting a cost, is small, otherwise it is large. Evaluating the local cost for each pair $\left(x_{n},y_{n}\right)$ one obtains the cost matrix $C\left(n,m\right):=c\left(x_{n},y_{n}\right);\;C\in {\mathbb{R}}^{N\times M}$. The best alignment between X and Y gives the minimum overall cost.

The total cost $c_{p}\left(X,Y\right)\;$ of a warping path between X and Y is defined by [27] as:

(8) $$c_{p}\left(X,Y\right)≔\;\overset{L}{\underset{\ell =1}{\sum }}c(x_{n_{\ell }},\;y_{m_{\ell }})$$

The optimal path between X and Y is the path with the minimum cost that meets boundary, continuity and monotonicity constraints. The first limitation means that the path starts at (1, 1) and ends at (*N, M*), the second says that only steps to adjacent elements of the matrix *C* are allowed, and the third limitation is that subsequent elements must be described by nondecreasing values of indexes *n, m*. The DTW(X,Y) distance between X and Y is defined as the total cost of $c_{p^{*}}$:

(9) $$DTW\left(X,Y\right)≔\;c_{p^{*}}\left(X,Y\right)=min\left\{c_{p}\left(X,Y\right)\;\right\}$$

The final value is obtained by dividing the value of DTW(X,Y) by the number of points on the track.

The dynamic programming method is used to determine the optimal path. In order to prevent undesirable situations, where a short fragment of one of the output runs will be matched to a long fragment of the second pass, an additional limit is introduced on the width of the so-called transformation windows that defines the search area as a set of cells in a narrow strip around the diagonal of matrix *C* connecting the beginning and ending elements of the path [27].

**Figure 3:**
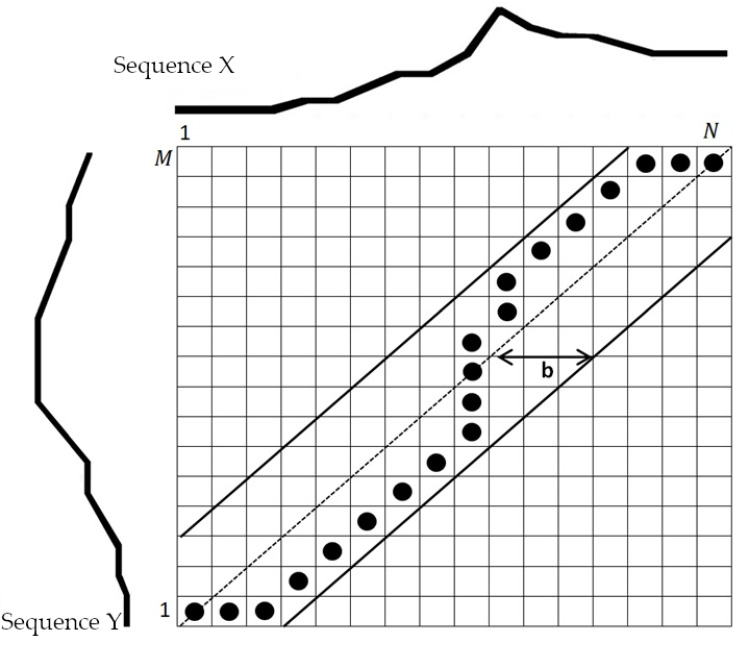
Visualization of the operation of the DTW algorithm with a transformation window width b.

Figure 3 presents the visualization of the operation of the DTW algorithm. A minimal transformation path was determined for two sequences X and Y. The transformation window with the width b has also been marked.

For each registered implementation of activities, characterized by a suitable time series, the DTW method was used to determine the values of similarities to other implementations of particular activities. In order to classify the test sample, the k-nearest neighbors classifier with k = 1, …,10 was used. Further details are explained in the following chapters.

### 4.2. BiLSTM

**Figure 4:**
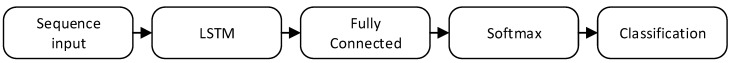
Long short-term memory (LSTM) network architecture.

The BiLSTM network is a modification of the long short-term memory (LSTM) network. The LSTM first used by Hochreiter and Schmidhuber in 1997 [28], is capable of learning long-term dependencies and is especially appropriate for classification of time series. It has a chain structure as shown in Figure 4 [29]. The sequence input layer introduces the data sequence or time series, the LSTM layer learns the long-term relationships between sequence time steps with its sophisticated structure which consists of a set of recurrently connected memory blocks, each with one memory cell and three multiplicative gates: input, output, and forget gate. The gates control the long-term learning of sequence patterns. During the training process each gate learns when to open and close, i.e., when to remember or forget information [29,30]. The prediction of class labels is presented in the classification layer which is preceded by a softmax layer and a fully connected layer.

**Figure 5:**
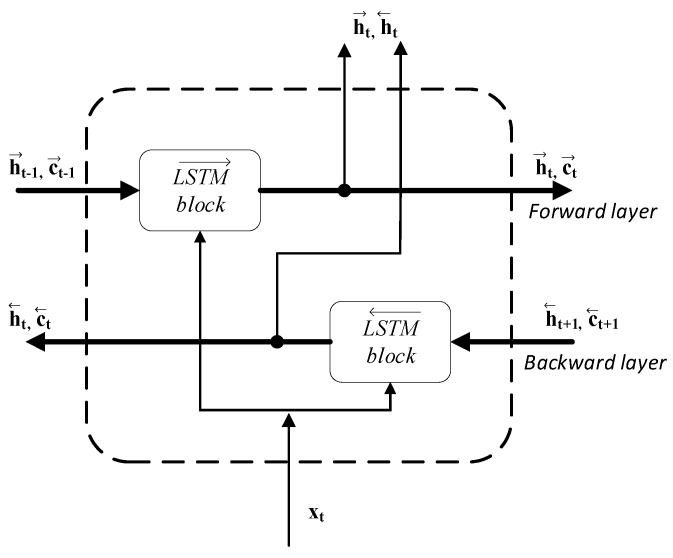
Bidirectional long short-term memory (BiLSTM) flow of data at time step t.

Unidirectional LSTM only maintains information of the past because the inputs it has seen are from the past. The BiLSTM, i.e., the bi-directional LSTM network processes one input from past to future (→) and one from future to past (←). In this way, for every point in a given sequence, the BiLSTM has complete information about all points before and after it. Flow of data at time step t  is shown in Figure 5.

The hidden state ${\vec{(h}}_{t},{\overset{\leftarrow }{h}}_{t})$ is the output of the BiLSTM layer at the time step t. The memory cell state ${\vec{c}}_{t-1}\;\left({\overset{\leftarrow }{c}}_{t+1}\right)\;$ contains information learned from the previous (subsequent) time steps. At each time step t, the forward layer and the backward layer add information to or remove information from the respective cell state, based on the actual step of the sequence $x_{t}$. The layers control these updates using gates, as mentioned earlier.

## 5. Datasets

In this work, we used two representative sets of data: UTD Multimodal Human Action Dataset and MSR-Action 3D Dataset.

### 5.1. UTD Multimodal HUMAN Action Dataset

**Figure 6:**
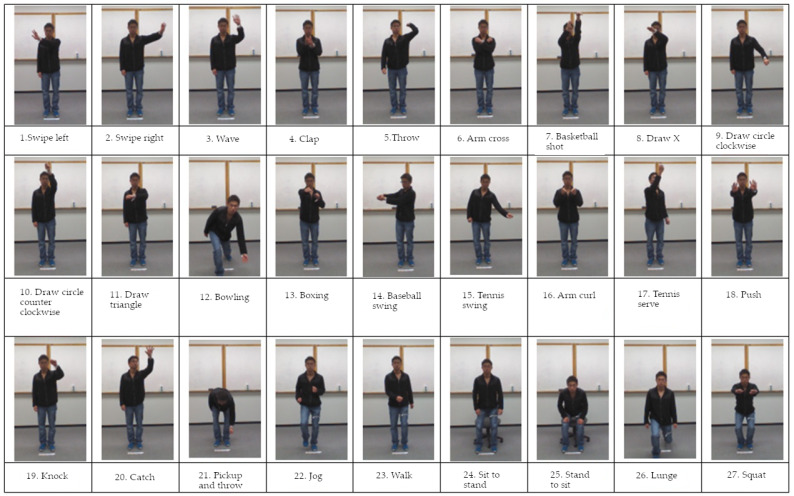
Activities in the University of Texas at Dallas Multimodal Human Action Dataset (UTD-MHAD).

UTD Multimodal Human Action Dataset (UTD-MHAD) is a publicly accessible database containing video sequences with registered behaviors and activities of people. The dataset consists of 27 activities performed by eight people (four women and four men). Each person repeats each action four times. After removing three damaged video sequences, the set contains 861 data samples. Activities in the database are presented in Figure 6. They can be divided into several categories, including sports activities (e.g., tennis service), gestures with hands (e.g., drawing the X sign), daily activities (e.g., knocking at the door) and exercising (e.g., squats) [9].

The data contained in the collection show large intra-class differences due to, inter alia, that: (i) people performed the same activities at different rates in different repetitions, (ii) people were of different height, (iii) activities were carried out naturally, so that each attempt is slightly different. An example may be clapping, where the number of claps in individual samples was different [6]. 

The set contains four data types for each sample. These are films: RGB, sequences of depth images, positions of skeletal joints of people (recorded by a Kinect^TM^ camera) and data from an inertial sensor placed on the body of people during the operation. For each repetition, RGB movies are saved in .avi files, depth image sequences, skeleton and data from the inertial sensor are stored in Matlab files format as three files with the .mat extension. The database is available in [9].

### 5.2. MSR-Action 3D Dataset

The MSR Action3D Dataset (MSR-Action 3D) contains 20 activities: high arm wave, horizontal arm wave, hammer, hand catch, forward punch, high throw, draw x, draw tick, draw circle, hand clap, two hand wave, side-boxing, bend, forward kick, side kick, jogging, tennis swing, tennis serve, golf swing, pickup & throw. Each activity is repeated two or three times by 10 people [10].

Also, in this set there are intra-class differences resulting from: (i) different speed of performing activities, (ii) different postures of individuals, (iii) how the activity is performed.

In the literature, among others in [31], the division of MSR into three subsets is applied:

- Action Set 1 (AS1): horizontal arm wave, hammer, forward punch, high throw, hand clap, bend, tennis serve, pickup & throw.
- Action Set 2 (AS2): high arm wave, hand catch, draw X, draw tick, draw circle, two hand wave, forward kick. side boxing.
- Action Set 3 (AS3): high throw, forward kick, side kick, jogging, tennis swing, tennis serve, golf swing, pickup & throw.

This division has also been used in the research carried out for this article.

The database is available in [10]. It contains sequences of depth images recorded by the Kinect^TM^ camera in the form of files with the extension .bin. A broader description of the dataset can be found in [11] and [5].

## 6. Activity Recognition System

The method can be described by following steps performed for each depth map frame:

(1) Segmentation of the human figure;
(2) Conversion of the depth map of the segmented human figure to the point cloud and downsampling the point cloud;
(3) Building the smallest possible axis aligned cuboid that entirely embraces the point cloud of the segmented human figure (bounding box);
(4) Dividing the bounding box into several cuboidal cells to increase the distinctiveness of features to be determined in the next steps;
(5) Computing the VFH descriptors for each cell and representing the histograms by their mean values *m*(.) and standard deviations *s*(.);
(6) Concatenation of the obtained *m*(.) and *s*(.) values into a feature vector.

Feature vectors related to frames of the movie constitute a time sequence which, after standardization (mean equal to zero, standard deviation equal to 1), represents the registered activity.

Segmentation is carried out to separate the human figure from the background elements so that the descriptor is determined only for it.

After segmentation, the depth map is converted into a point cloud. The coordinates of cloud points:$\;PC_{i}^{x}$, $PC_{i}^{y},$ and $PC_{i}^{z}$ were set with respect to the *DA* pixels’ depth value based on the perspective projection equations and Kinect^TM^ camera’s parameters [1]:

(10) $$PC_{i}^{x}=\frac{\left(DA_{i}^{z}+fl\right)*\left(\frac{DA_{width}}{2}-DA_{i}^{x}-1\right)*ps^{x}}{fl},$$

(11) $$PC_{i}^{y}=\frac{\left(DA_{i}^{z}+fl\right)*\left(\frac{DA_{height}}{2}-DA_{i}^{y}-1\right)*ps^{y}}{fl},$$

(12) $$PC_{i}^{z}=DA_{i}^{z},$$

where: $DA_{width}$ – the number of depth map columns, $DA_{height}$ – the number of depth map rows, fl – the Kinect^TM^ infrared camera’s focal length and $ps^{x}$ i $ps^{y}$ – the pixel dimensions, width and height, respectively.

In the case of datasets analyzed in this work, the data has been downloaded from the Kinect^TM^ camera whose parameters were given in [9] and [10] and set accordingly fl = 4.73 mm and $ps^{x}$= $ps^{y}$= 0.0078 mm. The resulting point cloud is redundantly dense. To reduce the number of points and to speed up the process of feature calculation the cloud is downsampled. This operation can be performed using PCL library.

After receiving point clouds, the process of extracting features using the VFH descriptor takes place using the PCL. The individual features of the descriptor consist of one histogram of size 45 bins. To avoid too much data, histograms are represented by their averages and standard deviations.

Before the classification stage, the received time series are standardized (mean values are zero, standard deviations 1) and compared with each other using the DTW method. The parameters of the method were chosen experimentally: distance measure - squared, width of the window - b = 6. The result of the DTW operation is the value of the distance between two runs, which is a measure of their similarity. In the last stage, the received DTW values are classified using the k-NN classifier with the parameter k = 1: 10. The second method used for classifying times series is the BiLSTM network described in Section 4.2.

## 7. Experiments

The details of the method described in the preceding section depend on the specific problem. Especially, this concerns dividing the bounding box into cells in point 4, feature selection in point 6, as well as classifier selection. These issues are discussed in this section.

The experiments described in this section are aimed at recognizing human activities, using only the information contained in the depth data.

The division of the datasets into training and testing was consistent with that adopted in literature. Basically, it was a leave-one-subject-out (LOSO) cross-validation, i.e., the dataset was divided into disjunctive subsets containing all actions presented by only one person and then one subject’s data was used as a test set in each fold of the cross-validation. 

**Figure 7:**
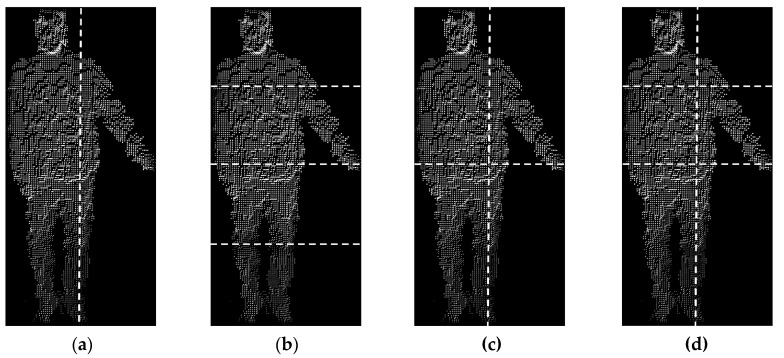
The considered decompositions of the person bounding box: (**a**) vertical division into two cells, (**b**) horizontal division into four cells, (**c**) cross-division into four cells, (**d**) division into six cells.

In order to increase the distinctiveness of the features of the VFH descriptor we decided to decompose the observed scene defined by a bounding box understood as a rectangular prism closely surrounding a point cloud describing the person silhouette. Four decompositions of the bounding box were considered: a) vertical division into two cells, b) horizontal division into four cells, c) cross-division into four cells, d) division into six cells (Figure 7).

A single element of the VFH descriptor consist of one histogram of size 45. As mentioned in Section 6, in this study each histogram is represented by the mean *m*(.) and standard deviation *s*(.). Thus, a single cell *i* of a particular video frame is represented by the eight-element feature vector:

(13) $$w_{i}=\left[m\left(\theta_{i}\right),s\left(\theta_{i}\right),m\left(\alpha_{i}\right),\;s\left(\alpha_{i}\right),\;m\left(\phi_{i}\right),s\left(\phi_{i}\right),\;m\left(d_{i}\right),\;s\left(d_{i}\right)\;\right]$$

and a bounding box divided into C cells is represented by the vector:

(14) $$w=\left[w_{1},w_{2},\ldots ,w_{C}\right],\;C\in \left\{1,\;2,\;4,\;6\right\}$$

**Figure 8:**
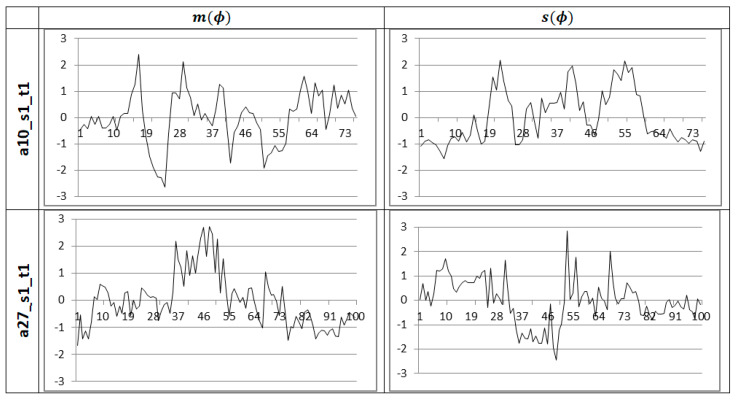
Sample standardized runs of mean and standard deviation for the ϕ feature of the VFH descriptor for two different activities.

Vectors (14) corresponding to a recorded sequence of frames form vector time series. For classification, each elementary time series of this vector time series is standardized. The videos in the UTD-MHAD have 45 – 125 frames (with average of 68), in the MSR-Action 3D 13-255 frames (with average of 41). The length of the time series affects the speed of classification. Sample standardized time series related to the VFH descriptor ϕ for two different activities, with the assumption that the bounding box is a single cell, are shown in Figure 8.

### 7.1. Activity Recognition Using DTW

Recognition results of LOSO validation with k-NN classifier for UTD-MHAD and MSR-Action 3D are presented in Table 2 and Table 3, respectively. The presented results confirm the legitimacy of the division of the bounding box into smaller cells, which positively influences the efficiency of activity recognition. For both datasets, the highest efficiency was obtained for the division into six cells, 88.58% (k = 10) for UTD-MHAD and 81.30% (k = 4) for MSR-Action 3D. In comparison with the case without division, there is a significant increase of the recognition efficiency. The division into six cells will be considered in the next experiments.

The obtained results are compared with available results presented in literature that refer to methods that also use the depth information only. Table 4 presents the comparison of the classification effectiveness of the proposed method and the method described in [6]. The authors of [6] also carried out the activity recognition using LOSO eight-fold cross-validation. The recognition rate obtained by the proposed method is 13.88 percent points higher.

Table 5 compares the recognition rates of the proposed method with the methods described in [6] and [32] where the authors carried out the recognition tests using realizations 1 and 2 of each activity as the training set and the remaining realizations as the test set. The recognition rate obtained by our method is higher by 14.2 points compared with [6] and by 6.04 points compared with [32].

The recognition rates for the MSR-Action 3D dataset were also compared with the results obtained by other authors. As mentioned in Section 5.2, this set has been divided into three subsets - AS1, AS2, AS3. Three tests were performed for each of the subsets. In the first test, “Test A”, $\frac{1}{3}$ of the data was used for training and the remaining $\frac{2}{3}\;$ for testing. In the second test, “Test B”, $\frac{2}{3}$ of the data was used for training, and $\frac{1}{3}$ for testing. In the last test, “Test C”, LOSO ten-fold cross-validation was used. Table 6 compares the recognition rates of the proposed method with the results obtained by Chen et al. [15] for the three tests.

The division of the bounding box into cells positively influenced the efficiency of classification. Nevertheless, the division was accompanied by a longer computation time due to the larger number of time series used by the algorithm. Table 7 presents a comparison of average classification times for the considered division of the bounding box obtained on a computer with the Intel Core i7-4702MQ, 2.2 GHz processor, k-NN and DTW performed with MATLAB R2019a.

The average classification times for the UTD-MHAD set are longer than for the MSR-Action 3D. This is due to the division of the MSR-Action 3D as described in Section 5.2 which reduces the size of the training set. Moreover, its videos are shorter which reduces computations required by the DTW algorithm.

The time needed for classification of time sequences with k-NN based on DTW depends mainly on the size of the training set as well as on the size of the feature vectors. We will try to reduce the values of these parameters while maintaining acceptable classification performance.

The first approach to shorten the time of classification was a reduction of the number of VFH histograms (of θ, α, ϕ, and d) used to create the feature vectors in (13). Two variants were considered: (V1) the feature vectors based on histograms of α, ϕ, and d, (V2) feature vectors based on histograms of  ϕ and d. The division of the bounding box into six cells was used for the research.

Table 8 presents the results for the UTD-MHAD. It shows that the reduction of the number of features does not significantly degrade the classification performance. For variant V1, the best recognition rate turned out even by 0.15 points higher, and for V2 by 0.08 points less than for the case without reduction.

Also for the MSR Action 3D dataset, the obtained classification results for the variant V1 are better than for the use of all features. For the V2 variant, the classification efficiency are also better than for the use of all features. The results are presented in Table 9.

Analyzing the classification times for particular variants and sets we observed the expected reduction of the classification time by a factor of ¼ (V1) and ½ (V2). Good classification efficiency has been preserved.

The second method of reducing the time of classification was limiting the training set to a certain number of representatives of each activity. For the UTD-MHAD two representatives of each activity were considered: (1) the median of the realizations of this activity in the training set by women and (2) the median of the realizations of this activity in the training set by men. For the MSR Action 3D it is difficult to determine the sex of a person, so the activity was represented by the median of its realizations in the training set. Determining of the median of a selected set of time series consisted in finding a series with the smallest sum of DTW distances between it and other series from this set.

The proposed two-step recognition algorithm using a set of representatives is as follows:

*Step 1*: The number k1 of the nearest neighbors of the classified activity is determined in a reduced training set composed of representatives of all activities.
*Step 2*: For a given number k = k2, the answer of the k-NN classifier is determined based on the training set containing all the realizations, i.e., before reduction, of the activities identified among k1 neighbors determined in step 1.

**Figure 9:**
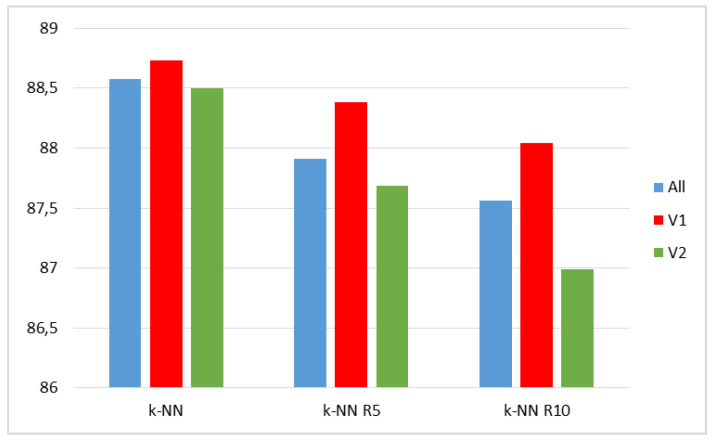
Recognition rates for the UTD-MHAD dataset: original k-NN (left) and k-NN using representatives.

The results of the test carried out on the UTD-MHAD dataset are presented in Table 10 and in Figure 9. Two values k1 = 5 and k1 = 10 were considered. For k1 = 5 the recognition rate in variant V1 of features was 0.47 points better, while in variant V2 it dropped around 0.22 points compared with the use of all features. For k1 = 10, the result in V1 proved to be 0.48 points better, and in V2 it was 0.57 points worse than in the case of using all features, for which, in turn, the result in the method of representatives turned out to be 1.02 points worse than in the original method, see Table 8.

**Figure 10:**
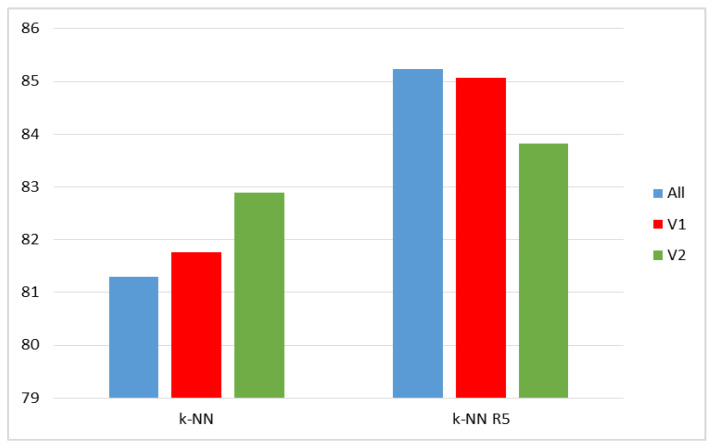
Recognition rates for the MSR Action 3D set: original k-NN (left) and k-NN using representatives.

For the MSR Action 3D set, due to the number of activities in the subsets AS1, AS2, and AS3, tests were performed using only k1 = 5. The results are presented in Table 11 and in Figure 10. The best results were obtained for using all the features, but the difference with variant V1 was only 0.17 points.

A summary of the best results for individual subsets is presented in Table 12.

Classification times using representatives were compared in Table 13.

Reducing the training set and the number of features gave very positive results. Taking into account the recognition rate, the preferred variant is V1. For this variant, also the time of classification is about 2.5 times shorter than in the case without reduction.

To assess these times, it is worth noting that the average time for determining the time series for a recognized activity based on a point cloud was about 480 ms for the UTD-MHAD dataset and about 289 ms for the MSR Action 3D dataset, and the average time needed for DTW based comparison of two series was, respectively, 0.5 ms and 0.3 ms.

### 7.2. Activity Recognition Using the BiLSTM Network

The research using the BiLSTM network was carried out using standardized sequences of activities derived from the division of bounding boxes into six cells. Based on the tests, the best network parameters were determined, allowing to obtain the best activity recognition efficiency. MATLAB R2019a software was used. The following parameter values were adopted: numHiddenUnits (number of hidden layers): 40 for the MSR 3D Action set and 125 for the UTD MHAD set, LearnRateDropFactor: 0.5, LearnRateDropPeriod: 20, dropoutLayer: 0.8, maxEpochs: 100, miniBatchSize: 1. The use of different number of hidden layers for individual datasets was conditioned by the length of time series describing the activities. Based on the conducted research, it was noticed that for longer time series the number of hidden layers must be correspondingly greater. This relationship has a significant impact on the effectiveness of network learning. Hence different values of the numHiddenUnits parameter were used for different datasets.

A kind of transfer learning was used in the research. The premise was to train a network and then use the obtained weights as starting weights in the next training of the same or other networks. The transfer learning was carried out three times for the UTD set, the results are presented in Table 14. The training was carried out in three cases: (i) using all features, (ii) using reduced features in variant V1, (iii) using reduced features in variant V2. Weight transfer had a positive impact on the increase in the effectiveness of the classification.

For the MSR 3D Action set a weight transfer was related with successive training on the subsets AS1, AS2, and AS3. The training path was as follows: AS1-> AS2 -> AS3 -> AS1-> AS2 -> AS3 -> AS1-> AS2 -> AS3. Details of the transfer and classification results are presented in Table 15. Such transfer of weights is simple, because the sets AS1, AS2, and AS3 include the same numbers of classes, as well as the structures and dimensions of respective networks trained on these sets are identical.

Similar research was also carried out for the LSTM network. The nature of the influence of weight transfer was similar, but the results were clearly worse, especially for the UTD-MHAD set, for which the time series are longer. Examined activities can be considered as sequences of interrelated elements. This probably justifies using the BiLSTM, which runs the inputs in two ways, one from past to future and one from future to past.

The big advantage of using the BiLSTM network is the short classification time equal, in average, to 14.8 ms for UTD-MHD (10.2 ms for MSR 3D Action), i.e., about 3 (1.5) times shorter compared with the k-NN based on DTW classifier. This encourages the use fusions of BiLSTM classifiers obtained at each of the three training stages characterized in Table 14 and Table 15. Various fusion methods are known, see e.g., [33]. One of the often used is the fuzzy integral method [33,34,35,36].

### 7.3. The use of BiLSTM Networks Fusion Using the Fuzzy Integral Method

The fusion was performed using three classifiers (BiLSTM networks) obtained in the LOSO test after first, second, and third training, independently for each of four datasets (AS1, AS2, AS3, and UTD-MHAD) and three feature vectors (all features, features in variant V1 and variant V2) using the following steps:

- The degree of importance $g^{i}$ of the classifier i, i∈{1, 2, 3} was determined as $g^{i}=p_{i}/\left(p_{1}+p_{2}+p_{3}\right)$ with $p_{i}$ denoting the recognition rate of the classifier *i* in the LOSO test.
- The fuzzy integral factor λ, λ ϵ (−1, +∞) was determined on the basis of the parameter $gm=\underset{i}{\max}g_{i}$ from the quadratic equation:
  (15) $$gm^{3}\lambda^{2}+3gm^{2}\lambda +\left(3gm-1\right)=0,$$
- Assuming that the output of the classifier i corresponding to a class k, k∈{1,2, …, K}  is $y_{i,k}\;$ and $Y_{k}=\left\{h_{1,k},\;h_{2,k},\;h_{3,k}\right\},$ $h_{1,k}\geq h_{2,k}\geq h_{3,k}\;$ where $h_{i,k}=y_{c\left(i\right),k}$, c(i)∈{1, 2, 3} specifies the classifier number, a class $\hat{k}\;$ resulting from the fusion of three classifiers was determined based on the relations:
  (16) $$G_{k}^{1}=g^{c\left(1\right)},\;\;G_{k}^{2}=g^{c\left(2\right)}+G_{k}^{1}+\lambda g^{c\left(2\right)}G_{k}^{1},\;\;G_{k}^{3}=g^{c\left(3\right)}+G_{k}^{2}+\lambda g^{c\left(3\right)}G_{k}^{2}$$
  (17) $$H_{k}=\underset{i}{\max}\left\{\min\left(G_{k}^{i},\;h_{i,k}\right)\right\}$$
  (18) $$\hat{k}=\arg\;\underset{k}{\max}\left(H_{k}\right)$$

**Figure 11:**
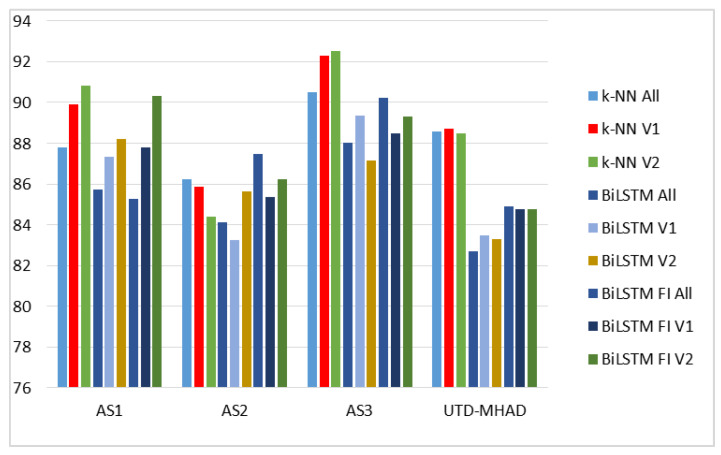
Comparison of the best recognition rates [%] obtained by various methods.

A summary of the best results obtained by various methods is shown in Table 16, where the three last columns are for the results obtained by fusion of BiLSTM classifiers, and in Figure 11.

## 8. Conclusions and Future Work

The article presents the use of depth image sequences for recognizing people’s activities. The subject of the research is a method using only depth information. The way of determining the features for classification is based on the use of the VFH point cloud descriptors of the 3D point clouds determined from respective depth maps. Three approaches to recognizing activities are considered: using the k-NN classifiers based on DTW measure, using the BiLSTM neural networks, using a fusion of the BiLSTM networks based on the fuzzy integral. Results of the classification experiments obtained for the representative, extensive datasets UTD-MHAD and MSR 3D are comparable or better to those known from the literature.

The contributions of this paper are: (i) introduction of a new method for human action recognition based on VFH point cloud descriptors; (ii) verification of the method on two representative, large datasets, (iii) reduction of the classification time for k-NN by a two tier approach, (iv) improvement of BiLSTM-based classification via transfer learning and combining multiple networks by the fuzzy integral.

In future work we plan to use additional point cloud descriptors, new classifiers, and datasets. Additional point cloud descriptors such as eigenvalue-based descriptors turned out to be beneficial for hand shape and fingerspelling recognition [1]. For this we consider the following classifiers: (a) the generalized mean distance-based k-NN classifier (GMDKNN) proposed in [37], where its advantage over the state-of-art k-NN-based methods is shown, (b) the two-phase probabilistic collaborative representation based-classification (TPCRC) [38] and the weighted discriminative collaborative competitive representation (WDCCR) [39] as new versions of the collaborative representation classifier CRC used in [6] for human action recognition, and (c) classifiers based on neural networks that directly use point clouds, e.g., [40].

## Figures and Tables

**Table 1:** Representative works using depth data for activity recognition.

Work	Method	Classifier	Dataset	Efficiency [%]
Wanging et al. [11]	*Action graph*: models the dynamics; its nodes (bag of 3D points) represent salient postures.	bi-gram with maximum likelihood decoding (BMLD)	MSR Action 3D *	AS1:72.9 AS2:71.9 AS3:79.2
Vieira et al. [12]	*Space–Time Occupancy Patterns*: space and time axes divided into segments define a 4D grid for each depth map sequence.	SVM	MSR Action 3D *	AS1:84.7 AS2:81.3 AS3:88.4
Wang et al. [13]	*Random Occupancy Patterns*: a sampling scheme that effectively explores very large sampling spaces; the features robustly encoded by sparse coding.	SVM	MSR Action 3D ** Gesture3D	86.2 88.5
Yang et al. [14]	*Depth Motion Maps*: accumulate sequences of depths and histograms of oriented gradients.	SVM	MSR Action 3D *	AS1:96.2 AS2: 84.1 AS3: 94.6
Chen et al. [6,15]	*Depth Motion Maps*: accumulate sequences of depths and histograms of oriented gradients.	Collaborative Representation classifier	MSR Action 3D *	AS1: 96.2 AS2: 83.2 AS3: 92
Oreifej et al. [16]	*Histogram of Oriented 4D Normals*: represents the distribution of the surface normal orientation in the space of time, depth, and spatial coordinates.	SVM	MSR Action 3D **	88.89
			MSR Hand Gesture	92.45
			3D Action Pairs	96.67
Kim et al. [17]	*Depth Motion Appearance, Depth Motion History*: local appearances and shapes are represented by histogram of oriented gradients.	SVM	MSR Action 3D *	90.45
Wanget al. [18]	*Joint Trajectory Maps*: the skeleton trajectory is projected to the three Cartesian planes and processed by three respective CNNs.	CNN	MSRC-12 Kinect Gesture *	93.12
			G3D Dataset *	94.24
			UTD-MHAD *	85.81
Kamel et al. [19]	*Depth Motion Image*: accumulates depth maps, *Moving Joints Descriptor*: represents motion of body joints; three CNN channels: DMI, (DMI+MJD), MJD.	CNN	MSR Action 3D * UTD-MHAD * MAD *	94.51 88.14 91.86
Hou et al. [20]	*Skeleton Optical Spectra*: encode the spatiotemporal information of a skeleton sequence into color texture images; desirable features are learned by three CNNs – for front, side, and top view.	CNN	MSR Action 3D * UTD-MHAD * MAD *	94.51 88.14 91.86
Wang et al. [21]	*3D Skeletons*: a joint descriptor (joint position, local space around it), *Fourier Temporal Pyramid* as a joint motion representation.	SVM	MSR Action 3D * MSRDailyActivity3D* CMU MoCap*	88.2 85.75 98.13
Yang, Tian, [22]	*EigenJoints*: features based on position differences of joints, combine static posture, motion, and offset.	Naïve-Bayes- Nearest-Neighbor Classifier	MSR Action3D *	AS1:74.5 AS2: 76.1 AS3: 96.4
Luo et al. [23]	*Temporal Pyramid Matching*: representation of temporal information in depth sequences; discriminative dictionary learning for sparse coding of the 3D joint features.	SVM	MSR Action3D **	96.7
			MSR DailyActivity 3D *	AS1: 97.2 AS2: 95.5 AS3: 99.1

CNN-Convolutional neural network, MAD-Multimodal action dataset, MHAD-Multimodal human action dataset, MSR- Mining Software Repositories, SVM-Support vector machine, UTD-University of Texas at Dallas. Datasets MSR (Action 3D and Activity 3d) are divided into three subsets AS1, AS2, and AS3. MSR Action 3D and UTD-MHAD are described in Section 5. * Protocol leave-one-subject-out ** First five subjects are used for training and the remaining five subjects are used for testing.

**Table 2:** Comparison of the recognition rates for the UTD-MHAD set, k-NN with DTW, and LOSO eight-fold cross-validation; the best values are marked in bold.

Number of the Nearest Neighbors	Recognition Rate [%]				
	Without Division	Vertical Division into 2 Cells	Cross Division into 4 Cells	Horizontal Division into 4 Cells	Division into 6 Cells
k = 1	74.78	80.24	82.34	85.48	86.37
k = 2	72.80	81.52	81.99	83.37	84.20
k = 3	75.59	81.40	82.10	85.59	86.63
k = 4	76.99	80.82	84.19	86.75	87.56
k = 5	78.96	80.83	83.97	87.10	88.15
k = 6	79.43	83.26	83.96	87.42	88.03
k = 7	79.08	82.91	83.27	87.55	86.98
k = 8	78.50	83.02	83.15	**88.49**	86.64
k = 9	**79.77**	83.37	**85.01**	87.67	87.57
k =10	79.43	**83.60**	84.54	87.90	**88.58**

**Table 3:** Comparison of the recognition rates for the MSR-Action 3D set, k-NN with DTW, and LOSO ten-fold cross-validation.

Number of the Nearest Neighbors	Recognition Rate [%]				
	Without Division	Vertical Division into 2 Cells	Cross Division into 4 Cells	Horizontal Division into 4 Cells	Division into 6 Cells
k = 1	59.56	69.66	69.75	69.20	77.09
k = 2	52.55	67.89	68.68	64.66	75.42
k = 3	58.07	73.89	73.63	69.91	79.78
k = 4	60.41	71.56	73.15	70.28	**81.30**
k = 5	59.93	72.30	73.90	71.16	81.19
k = 6	61.21	74.96	73.15	69.21	80.51
k = 7	63.53	74.73	73.60	71.20	81.05
k = 8	63.54	75.49	73.61	70.40	80.08
k = 9	62.87	75.68	**74.90**	70.85	80.29
k =10	**64.62**	**77.09**	74.62	**71.92**	79.43

**Table 4:** Comparison of the recognition rates for the UTD-MHAD set and LOSO eight-fold cross-validation.

Method	Recognition Rate [%]
Wang et al. [18]	85.81
Hou et al. [20]	86.97
Kamel et al. [19]	88.14
Our work	**88.58**

**Table 5:** Comparison of the recognition rates for the UTD-MHAD set and realizations 1 and 2 in the training set and 3, 4 in the test set.

Method	Recognition Rate [%]
Chen et. al. [6]	85.10
Mandany et. al. [32]	93.26
Our work	**99.30**

**Table 6:** Comparison of the recognition rates for the MSR-Action 3D dataset.

Data Set	Chen et al. [15]			Proposed Method		
	Test A	Test B	Test C	Test A	Test B	Test C
AS1	97.3	98.6	96.2	100	95.3	87.8
AS2	96.1	98.7	83.2	94.9	93.5	86.2
AS3	98.7	100	92	100	94.7	90.5
Average	97.4	**99.1**	**90.5**	**98.3**	94.5	88.1

**Table 7:** Classification time.

Division of the Bounding Box	Average Classification Time [ms]	
	UTD-MHAD	MSR-Action 3D
Without division	81.2	38.8
Vertical division into 2 cells	86.0	41.3
Cross division into 4 cells	95.5	52.5
Horizontal division into 4 cells	97.1	53.2
Division into 6 cells	112.9	57.4

**Table 8:** Recognition rates for the UTD-MHAD dataset and two variants of feature reduction (eight-fold cross-validation LOSO).

Number of the Nearest Neighbors	Recognition Rate [%]		
	All Features (Based on [θ, α, ϕ,d])	V1: Features Based on [α,ϕ, d]	V2: Features Based on [ϕ, d]
k = 1	86.37	86.28	85.59
k = 2	84.20	84.20	85.13
k = 3	86.63	87.80	85.71
k = 4	87.56	87.45	86.52
k = 5	88.15	87.92	87.23
k = 6	88.03	87.68	87.34
k = 7	86.98	87.46	87.45
k = 8	86.64	**88.73**	88.39
k = 9	87.57	87.92	**88.50**
k = 10	**88.58**	87.92	87.92

**Table 9:** Recognition rates for the MSR Action 3D dataset and two variants of feature reduction (ten –fold cross-validation LOSO).

Number of the Nearest Neighbors	Recognition Rate [%]		
	All Features (Based on [θ, α, ϕ, d])	V1: Features Based on [α,ϕ,d]	V2: Features Based on [ϕ,d]
k = 1	77.09	80.21	79.03
k = 2	75.42	75.40	75.66
k = 3	79.78	78.84	80.97
k = 4	**81.30**	79.96	80.56
k = 5	81.19	79.79	81.45
k = 6	80.51	79.65	**82.89**
k = 7	81.05	79.87	82.10
k = 8	80.08	80.13	80.45
k = 9	80.29	**81.77**	82.09
k =10	79.43	72.46	82.62

**Table 10:** Recognition rates for the UTD-MHAD and k-NN using representatives (eight-fold cross-validation LOSO).

Number of the Nearest Neighbors k2	Recognition Rate [%]					
	k1 = 5			k1 = 10		
	All Features	V1	V2	All Features	V1	V2
1	85.94	86.28	86.29	85.24	86.40	85.59
2	84.90	85.36	85.71	84.43	85.24	85.12
3	86.63	87.80	85.71	86.51	87.21	85.24
4	86.87	87.46	87.22	87.10	87.33	85.71
5	**87.91**	**88.38**	87.46	87.45	**88.04**	86.18
6	87.45	87.46	87.57	**87.56**	87.57	86.64
7	86.64	88.03	86.76	86.52	86.53	86.06
8	85.59	87.33	87.11	86.87	87.57	86.76
9	86.98	86.76	**87.69**	86.99	86.87	**86.99**
10	86.87	87.45	86.99	86.98	86.41	86.87

**Table 11:** Recognition rates for the MSR Action 3D dataset using representatives (ten-fold cross-validation LOSO).

Number of the Nearest Neighbors k2	Recognition Rate [%], k1 = 5		
	All Features	V1	V2
1	83.12	83.07	83.05
2	83.86	80.86	81.18
3	**85.23**	83.94	83.19
4	84.64	84.32	82.05
5	84.82	**85.06**	83.45
6	84.55	84.50	83.45
7	84.55	84.28	**83.83**
8	83.74	84.66	83.78
9	84.32	85.00	83.18
10	83.95	84.87	83.45

**Table 12:** Recognition rates [%] for the MSR Action 3D set and k-NN using representatives - best results for AS1, AS2, AS3 subsets (ten-fold cross-validation LOSO).

AS1			AS2			AS3		
All Features	V1	V2	All Features	V1	V2	All Features	V1	V2
84.4	87.0	**88.2**	**82.6**	82.5	80.9	**91.7**	89.6	86.0

**Table 13:** Comparison of average classification times for tested sets and k-NN using representatives.

Variants of the Features	Average Classification Time Using Representatives [ms]		
	UTD-MHAD		MSR Action 3D
	k1 = 5	k1 = 10	k1 = 5
All features	46.2	59.1	22.5
V1	40.9	53.3	16.9
V2	37.9	49	11.3

**Table 14:** Recognition rates [%] obtained using weight transfer in the Bidirectional long short-term memory (BiLSTM) network for the UTD-MHAD set (eight-fold cross-validation LOSO).

	Training 1 (Random Starting Weights)	Training 2 (Starting Weights Based on Training 1)	Training 3 (Starting Weights Based on Training 2)
All the features	80.70	82.68	82.45
Variant V1	80.24	83.14	84.48
Variant V2	81.98	83.27	82.33

**Table 15:** Recognition rates [%] obtained using weight transfer in the BiLSTM network for the MSR 3D Action (ten-fold cross-validation LOSO) – all features/features in variant V1/ features in variant V2.

First Training	Second Training	Third Training
AS1 (random starting weights)	AS1 (starting weights taken from the first training on AS3)	AS1 (starting weights taken from the second training on AS3)
83.60/83.58/88.22	85.19/87.31/86.59	85.72/86.89/87.73
AS2 (starting weights taken from the first training on AS1)	AS2 (starting weights taken from the second training on set AS1)	AS2 (starting weights taken from the third training on AS1)
83.43/83.24/85.63	81.16/82.43/82.41	84.11/82.01/8455
AS3 (starting weights taken from the first training on AS2)	AS3 (starting weights taken from the second training on AS2)	AS3 (starting weights taken from the third training on AS2)
87.64 /87.24/86.27	86.90/87.51/84.62	88.03/89.34/87.14

**Table 16:** Comparison of the best recognition rates [%] obtained by various methods (LOSO cross-validation).

Title 1	k-NN			BiLSTM			BiLSTM + Fuzzy Integral		
	All Features	V1	V2	All Features	V1	V2	All Features	V1	V2
AS1	87.80	89.89	**90.83**	85.72	87.31	88.22	85.26	87.77	90.34
AS2	86.23	85.85	84.40	84.11	83.24	85.63	**87.48**	85.36	86.23
AS3	90.5	92.28	**92.50**	88.03	89.34	87.14	90.21	88.49	89.30
UTD-MHAD	88.58	**88.73**	88.50	82.68	83.48	83.27	84.89	84.77	84.77
